# Brain White Matter Alterations in Young Adults with Childhood Emotional Neglect Experience

**DOI:** 10.3390/bs15060746

**Published:** 2025-05-28

**Authors:** Xiaokang Jin, Bin Xu, Hua Jin, Shizhen Yan

**Affiliations:** 1 Faculty of Psychology, Tianjin Normal University, Tianjin 300387, China; jinxiaokang@stu.tjnu.edu.cn; 2 Student Mental Health Education and Counseling Center, Tianjin University of Science & Technology, Tianjin 300457, China; tjkdxubin@tust.edu.cn; 3 Academy of Psychology and Behavior, Tianjin Normal University, Tianjin 300387, China; 4 School of Health, Fujian Medical University, Fuzhou 350122, China

**Keywords:** childhood emotional neglect, childhood trauma, white matter, automating fiber tract quantification (AFQ), fractional anisotropy (FA)

## Abstract

Childhood trauma encompasses various subtypes, and evidence suggests that neurodevelopmental damage differs across these subtypes. However, the specific impact of childhood emotional neglect (CEN), a distinct subtype of childhood trauma, on the microstructural integrity of brain white matter remains unclear. Therefore, the present study aims to investigate the effects of CEN on the microstructure of brain white matter in young adults using diffusion tensor imaging. After administering online questionnaires, conducting interviews, and obtaining diagnoses from specialized physicians, we recruited 20 young adults with a history of CEN and 20 young adults with no history of childhood trauma. Using automating fiber tract quantification (driven by a diffusion tensor model), we traced the 20 primary white matter fibers and divided each fiber into 100 nodes for analysis. Group differences in fractional anisotropy (FA) at each node of each fiber were then examined. The results revealed that the FA values at nodes 1–35 of the right thalamic radiation were consistently lower in the emotional neglect group compared to the control group (after FEW correction, cluster threshold = 22, p-threshold = 0.005). These findings suggest an association between CEN and reduced FA values in the right thalamic radiation, indicating alterations in brain white matter. Overall, our results contribute to the theoretical understanding of how “experience shapes the brain,” providing new insights into the neurostructural consequences of childhood emotional neglect.

## 1. Introduction

Childhood emotional neglect (CEN) pertains to the failure to meet a child’s fundamental emotional needs, insensitivity towards their distress, and disregard for their emotional and social development (Teicher & Samson, 2013); it is one subtype of childhood trauma. Most research has concentrated on active forms of trauma—such as sexual and physical abuse—whereas passive, more covert emotional neglect has received comparatively little attention (Stoltenborgh et al., 2013). The findings of meta-analyses indicate a considerably high global prevalence of CEN, estimated to be approximately 18% (Stoltenborgh et al., 2013, 2015). Furthermore, CEN also frequently occurs independently of other childhood trauma subtypes (emotional neglect only), with a separate prevalence of 6.2% (Taillieu et al., 2016). Recent research findings indicate that CEN has enduring negative implications for mental health. A history of CEN is linked to various negative outcomes, including but not limited to depression, anxiety, stress, and substance abuse (Grummitt et al., 2022; Infurna et al., 2016; Salokangas et al., 2020). Due to its covert character, high prevalence, and serious mental health risks, it is crucial to study the long-term, specific effects of CEN. This research facilitates the prevention of and intervention in negative problems secondary to CEN, and it may provide a unique perspective on how early experiences shape human beings.

Given the close association of CEN with mental illness, an increasing number of studies have started to focus on the specific effects of CEN on the brain. Recently, neuroimaging studies have found significant differences between adults with and without CEN in gray matter volumes in the bilateral amygdala and hippocampus (Womersley et al., 2020), brain activation when performing social exclusion or face processing tasks (Schulz et al., 2022; White et al., 2012), and resting-state functional connectivity patterns (X. Jin et al., 2023; B. Xu et al., 2023). These studies indicate that CEN does have a detrimental impact on brain development. However, far less is known about the long-term effect of CEN on the brain’s white matter structure, which is crucial for advancing our comprehension of the pathogenesis of psychological and behavioral problems associated with CEN.

White matter tracts connect various brain regions to form structural networks and facilitate effective neural communication. White matter structures are continuously refined and remodeled throughout childhood by a combination of genetic and environmental conditions, laying an important foundation for well-developed cognitive abilities and mental health (Lebel & Deoni, 2018). Diffusion tensor imaging (DTI) is an advanced, non-invasive technique of magnetic resonance imaging (MRI) that enables visualization as well as qualitative and quantitative assessment of the microstructure integrity of the white matter tracts (Chanraud et al., 2010). Using DTI techniques, there is growing evidence that general childhood trauma (without distinguishing between subtypes of trauma) alters the white matter integrity (Asmal et al., 2019; Costello et al., 2023; He et al., 2023; H. Huang et al., 2012; Lim et al., 2020; Lu et al., 2013). For example, healthy young adults with childhood trauma (without differentiating between specific trauma subtypes) exhibited significantly lower fractional anisotropy (FA) in the anterior thalamic radiation, right posterior corona radiata, left super corona radiata, right anterior corona radiata, and right posterior limb of the internal capsule (He et al., 2023). The first meta-analysis showed that individuals with childhood trauma (without differentiating between specific trauma subtypes) show significantly reduced FA in the optic radiations, left anterior thalamic radiation and bilateral fornix, inferior frontal-occipital fasciculus, and inferior longitudinal fasciculus, along with the anterior portions of the corpus calltosum (Lim et al., 2020). Additionally, studies focusing on children in early neglect settings, such as orphanages or foster homes, provide indirect evidence that childhood trauma may affect the integrity of white matter (Govindan et al., 2010; Hanson et al., 2013). For example, Hanson et al. (2013) found that children in institutional care had lower white matter directional organization in the PFC and lower directional organization in white matter tracts connecting the temporal lobe and the PFC.

However, none of the above studies distinguished the various subtypes of childhood trauma. Moreover, the mechanisms by which different childhood trauma subtypes increase the risk of neurodevelopmental deficits and psychiatric disorders are distinctly different. Approaches that do not distinguish between subtypes of childhood trauma make it difficult to reveal the unique effects of each subtype on neurodevelopment. The early stress model viewed all childhood trauma subtypes as the same stressor, and they shared a common pathogenic mechanism (Neigh et al., 2009; Teicher et al., 2003; Teicher & Samson, 2016). It has been shown that chronic exposure to stress can lead to abnormal hypothalamus–pituitary–amygdala (HPA axis) functioning, resulting in excessive or blunted glucocorticoid release, as well as structural consequences in the brain (McEwen, 2012). However, McLaughlin et al. (2014) argued that the history of childhood trauma could not be explained simply in terms of stress. Childhood traumas were further categorized into threatening types (i.e., the presence of an experience that poses a threat to a person’s physical integrity) and deprivation types (i.e., the lack of expected environmental input and complexity). Under this dimension, physical, emotional, and sexual abuse are considered a high-threat but low-deprivation experience, whereas neglect is a high-deprivation, low-threat experience. According to McLaughlin et al. (2014), early exposure to threatening environments can lead to long-term changes in neural circuits related to emotional learning.

Recent neuroimaging studies show that different types of childhood trauma have varying long-term impacts on brain structure (Cassiers et al., 2018; Hendrikse et al., 2024). Hendrikse et al. (2024) found that non-violent/deprivation trauma specifically leads to reduced FA in the bilateral, left, and right anterior limbs of the internal capsule (ALIC). However, the specific effects of childhood emotional neglect (CEN) are still unclear. Additionally, behavioral results also suggest that the pathogenic mechanisms of CEN for some psychiatric disorders are distinct from other subtypes. In comparison with other subtypes of childhood trauma, CEN predicts significantly more anxiety, depression, stress, alexithymia, resilience, and substance abuse disorders (Aust et al., 2013; Grummitt et al., 2022; Salokangas et al., 2020). For example, CEN is the only subtype of childhood trauma linked to alexithymia, a trait that makes identifying and communicating feelings difficult (Aust et al., 2013). Moreover, research by Ho and Schermer (2024) indicates that emotional neglect is the most robust predictor of loneliness among the various subtypes of childhood trauma. In addition, Grummitt et al. (2022) demonstrated that emotional neglect is distinctly linked to depression, anxiety, and stress, whereas physical neglect does not exhibit such associations. These findings suggest that the pathogenic mechanisms underlying emotional neglect may differ significantly from those associated with other trauma subtypes, highlighting its unique contribution to mental health disorders.

Taken together, these findings led us to speculate that mixing different forms of childhood trauma into one may obscure their unique impacts on neurodevelopment. It is necessary to explore the influence of CEN on white matter structure separately. The primary aim of the current study was to reveal the specific effect of CEN on the microstructure of the brain white matter by comparing the differences in the white matter integrity of the brain between healthy young adults with CEN only and without any subtype of childhood trauma. We hypothesized that white matter integrity would be lower in young adults with CEN only than in controls.

## 2. Materials and Methods

### 2.1. Participants

The subject screening and evaluation process is described below: First, we conducted an online preliminary assessment of childhood trauma using the Childhood Trauma Questionnaire Short Form (CTQ-SF) with 5010 college students from four colleges and universities in Tianjin. The CTQ-SF is a self-report questionnaire that is widely used to assess traumatic childhood experiences (Bernstein et al., 2003). The Chinese version of the current study had good reliability and validity (Zhao et al., 2005). Second, at least one month later, potential participants eligible for initial screening were recruited again for online interviews, CTQ-SF retesting, and psychiatric disorder diagnosis. The CTQ retest was designed to ensure the reliability of the participant screening to minimize the impact of participants misreading the questionnaire and recall bias on the results. Two clinical professionals conducted mental disorder diagnoses using the Structured Clinical Interview for DSM-IV Axis I Disorders (SCID-I/P) to ensure that no participants met criteria for any additional psychiatric disorders. Finally, participants who met the inclusion criteria were invited to participate in subsequent MRI experiments. All participants in the experiment provided informed consent. The local ethics committee (Tianjin Normal University Ethics Committee) granted the experiment ethical approval.

Using the cut-off scores of moderate-to-severe childhood trauma, as recommended by relevant studies, the significance of each CTQ-SF factor was determined; a score below the cut-off is correspondingly considered non-trauma (Kim et al., 2018; Peters et al., 2019; Sáez-Francàs et al., 2015; Wu et al., 2020). Consequently, individuals who fulfilled the cut-off of CTQ-SF, indicating a predominant experience of childhood emotional neglect, were identified and categorized as members of the childhood emotional neglect (CEN) group. Accordingly, individuals who meet the following assessment criteria were included in the CEN group, indicating that they were primarily affected by childhood emotional neglect: scores for physical abuse < 9, emotional abuse < 12, sexual abuse < 7, physical neglect < 9, and only emotional neglect ≥ 15. Additionally, participants with the lowest score of 5 for all childhood trauma subtypes on the CTQ-SF assessment should be considered not to have suffered any childhood trauma and be included in the control group.

Twenty young adults with a CEN history and twenty controls without any trauma history were recruited in this study. One CEN participant and one control participant were excluded from the formal analysis due to incomplete data. Thus, in total, 19 CEN participants (9 females, 19.3 ± 0.89 years) and 19 control participants (9 females, 19.9 ± 1.1 years) were included in the analysis.

### 2.2. Data Acquisition

Brain images of all participants were acquired using a Siemens Prisma 3.0T MRI scanner with a 64-channel head coil at the Brain Imaging Center of Tianjin Normal University. DTI diffusion image: repetition time (TR) = 8500 ms, echo time (TE) = 63 ms, slice thickness = 2.0 mm, slice number = 75, field of view (Fov) = 224 mm × 224 mm, matrix = 112 × 112, no gap between slices, imaging time = 363 s, voxel size = 2 × 2 × 2 mm^3^. DTI data were acquired at 10 b-values of 0 s/mm^2^ and a single b-value of 1000 s/mm^2^ with 64 diffusion encoding directions chosen to be approximately isotropically distributed on a full sphere based on the electrostatic repulsion model.

T1-weighted 3-D structural image: repetition time (TR) = 2530 ms, echo time (TE) = 2.98 ms, slice thickness = 1 mm, field of view (Fov) = 256 mm × 256 mm, matrix = 256 × 256, inversion time = 1100 ms, flip angle (FA) = 7°, slice number = 192, voxel size = 1 × 1 × 1 mm^3^.

### 2.3. Automatic Fiber Tract Quantification (AFQ) Analysis

In this study, data preprocessing and automated fiber quantification (AFQ) analysis were conducted using the NeuroScholar cloud platform (http://www.humanbrain.cn, Beijing Intelligent Brain Cloud, Inc., Beijing, China). This platform employs the Python-based QSIPrep pipeline for preprocessing diffusion tensor imaging (DTI) data (Cieslak et al., 2021; the source code is available at https://github.com/pennbbl/qsiprep, accessed on 1 May 2024) and utilizes the Python-based AFQ package for the automated quantification of fiber tracts (Yeatman et al., 2012; the open-source AFQ toolbox is available at https://github.com/yeatmanlab/AFQ, accessed on 1 May 2024).

QSIPrep leverages BIDS metadata to automatically set up a suitable preprocessing workflow (Cieslak et al., 2021). The steps include: (1) conforming image and gradient orientation; (2) grouping images by phase encoding for motion and distortion correction; (3) denoising diffusion-weighted images with MP-PCA or patch2self; (4) correcting distortions; (5) correcting head motion; (6) creating a subject b = 0 template; and (7) registering the b = 0 reference image to the skull-stripped T1w image. For more details, see the QSIPrep documentation (Cieslak et al., 2021).

To fit the tensors, a robust least-squares algorithm was employed, which is designed to exclude outliers during the tensor estimation (Chang et al., 2005). The eigenvalues obtained from the diffusion tensor’s decomposition were used to compute the fractional anisotropy (FA). Our analysis concentrated primarily on FA values, given that preliminary evidence in the literature associates emotional neglect with alterations in FA (Hendrikse et al., 2024; Meinert et al., 2019). Furthermore, FA values are the most frequently and independently used metrics in AFQ analyses (Daood et al., 2025; L. Huang et al., 2021; F. Xu et al., 2022).

Then, automatic fiber tract quantification (AFQ) was conducted, which involved the following steps: (1) Whole-brain fiber tracts were estimated using a deterministic streamline tracking algorithm (STT) with FA thresholds over 0.2 and turning angles under 30 degrees (Mori et al., 1999). (2) Fiber tracts were segmented via the waypoint ROI method (Wakana et al., 2007). (3) Probabilistic fiber tract maps refined the tracts (Hua et al., 2008). (4) An iterative method was used to clean the tracts by removing fibers exceeding four standard deviations in length or deviating from the core. (5) FA values were quantified at 100 nodes along each fiber bundle, represented as a vector of 100 values.

### 2.4. AFQ Statistical Analysis

For AFQ analysis, intergroup comparisons were performed using SPSS.24 statistical software. Independent-sample *t*-tests were performed on the FA values of 100 points for each fiber separately, with a significance level of *p* < 0.05. Multiple comparisons were adjusted using cluster-level family-wise error (FWE) correction at *p* < 0.05. The FWE determines the cluster size threshold through an internal permutation test based on the specified alpha value (Nichols & Holmes, 2001). Fiber bundles with consecutive significant nodes exceeding this threshold are considered significant, indicating intergroup differences (Yeatman et al., 2012; the code for the AFQ multiple-comparison correction can be accessed at https://github.com/yeatmanlab/AFQ/blob/master/functions/AFQ_MultiCompCorrection.m, accessed on 1 May 2024).

### 2.5. Machine Learning Analytics—Support Vector Machines (SVMs)

After identifying white matter bundles with significant intergroup differences, we used a Support Vector Machine (SVM) classifier to evaluate the effectiveness of features from these bundles in diagnosing emotional neglect. This methodology not only corroborates the reliability of the *t*-test results but also underscores the critical role of white matter architecture in predicting emotional neglect, thereby providing potentially valuable insights for clinical diagnosis.

We used the significance results of two-sample *t*-tests as raw features of the SVM (Wang et al., 2022), and the raw feature matrix included 100 FA values for each participant (Chen et al., 2023). We used a nested cross-validation approach to optimize and evaluate the performance of classification models. Specifically, two loops are implemented in nested cross-validation, the inner and outer loops. The inner loop uses the grid search method to obtain the optimal hyperparameters C and γ of the model. The outer loop then uses the optimal hyperparameters to evaluate the performance of the model. The nested cross-validation is effective in preventing data leakage so that the resulting test set error is almost the true error (Varma & Simon, 2006). Leave-one-out cross-validation was used to estimate classification error in both inner and outer loops. The receiver operating characteristic (ROC) curve was performed to evaluate SVM models, and the area under the curve (AUC) was calculated to quantify the ROC. Model performance evaluation was based on metrics such as accuracy, sensitivity, specificity, and precision. Furthermore, to assess if the classification performance was better than chance, we conducted a permutation test by randomly shuffling class labels in the training data and performing cross-validation on this permuted set 1000 times. We then calculated the proportion of permuted accuracy values that matched or exceeded the actual accuracy. If fewer than 5% (*p* < 0.05) of these values surpassed the actual accuracy, it was deemed statistically significant.

## 3. Results

**Figure 1:**
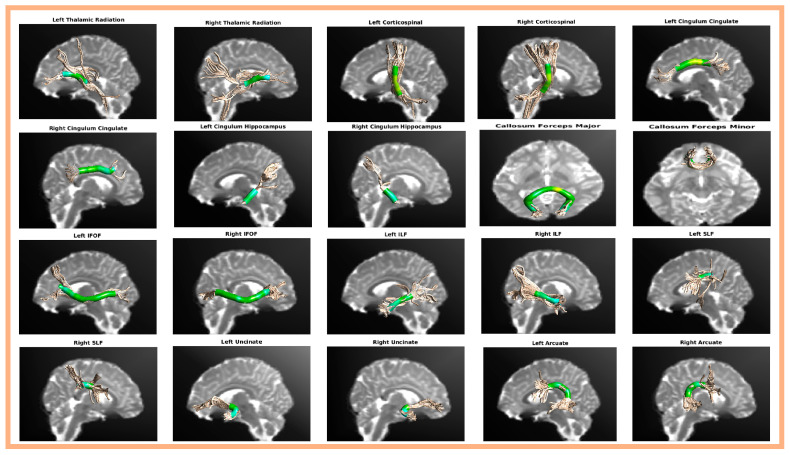
Schematic of the anatomy of the 20 major fiber bundles identified using AFQ analysis.

AFQ successfully identified 20 principal fiber bundles, including the bilateral thalamic radiation, bilateral corticospinal, bilateral cingulum cingulate, bilateral cingulum hippocampus, callosum forceps major, callosum forceps minor, bilateral inferior frontal-occipital fasciculus, bilateral inferior longitudinal fasciculus, bilateral superior longitudinal fasciculus, bilateral uncinate, and bilateral arcuate. The anatomical locations of the 20 fiber bundles reconstructed by AFQ are shown in Figure 1.

**Figure 2:**
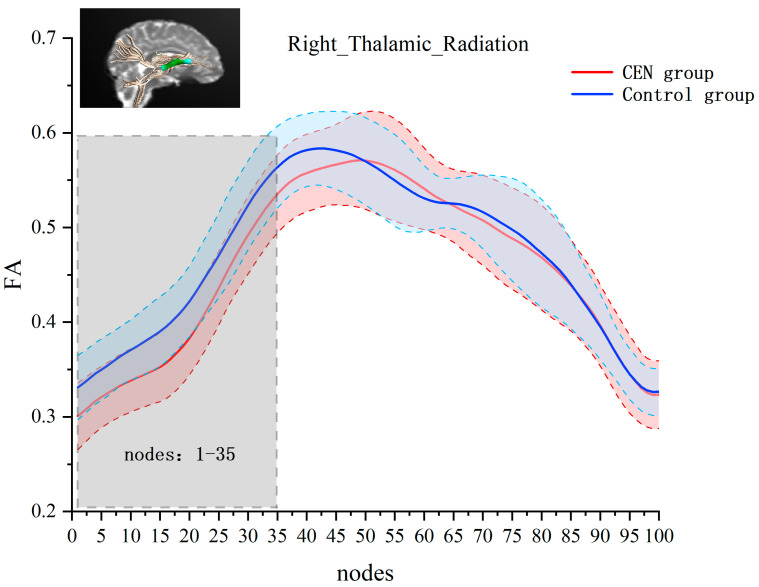
Analysis of FA values at 100 nodes on the right thalamic radiation bundle for the emotional neglect and control groups. The red line and the red area indicate the mean and standard deviation of FA for the emotional neglect group at that node. The blue line and blue area indicate the mean and standard deviation of FA for the control group at that node. Horizontal axis coordinates indicate 100 equal nodes of the right thalamic radiation bundle, and vertical axis coordinates indicate FA values. Gray areas are areas with significant differences between groups of FA values.

Next, we performed independent-samples *t*-tests for FA values at each node of each fiber bundle separately. The results showed significant between-group differences in the right thalamic radiation. Compared to the control group, the emotionally neglected group had lower FA values at nodes 1–35 of the right thalamic radiation (after FEW correction, cluster threshold = 22, p-threshold = 0.005) (see Figure 2).

**Figure 3:**
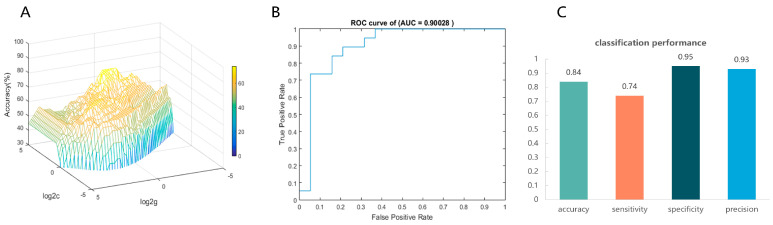
Visualization of SVM classification performance. (**A**) The optimal hyperparameters were determined using the grid search method. The hyperparameter values are on a log scale; specifically, log2C and log2γ are searched in the range [−5 5] in steps of 0.2 (best c = 18.3792, best γ = 0.7579). (**B**) The receiver operating characteristic curve (ROC) was employed for classification using the optimized model, and the area under ROC curve (AUC) was used as a measure of the predictive performance of our SVM models. (**C**) SVM model’s accuracy, sensitivity, specificity, and precision.

Then, each participant’s FA values for 100 nodes of the right thalamic radiation bundle were used as raw features to train and subsequently classify the CEN group and the control group. In the inner loop, the grid search reveals that the model had the best classification efficacy under the optimal hyperparameters C = 18.3792, γ = 0.7579 (see Figure 3A). In the outer loop, the optimized model exhibited a good classification performance. The AUC value = 0.90028, which indicates good discrimination (see Figure 3B). Meanwhile, the optimal model predicted accuracy = 0.84, sensitivity = 0.74, specificity = 0.95, and precision = 0.93, indicating that the model has a good predictive performance (see Figure 3C). The 1000-permutation test revealed a mean accuracy of 78.95% for non-permutation and a *p*-value of 0.006 after permutation, indicating statistically significant classification accuracy above random chance.

## 4. Discussion

Previous research has extensively documented the profound psychological and physiological harm caused by childhood trauma (Liu et al., 2023; Moreira et al., 2024). However, the unique contribution of CEN remains unknown. The present study is the first to explore CEN’s unique contribution to the microstructure of brain white matter.

AFQ analysis showed that right thalamic radiation fractional anisotropy (FA) was significantly lower in the CEN group compared to the control group. Decreased FA implies a decrease in white matter integrity, resulting from a poor degree of myelination, poor fiber density, high permeability, or enlarged extracellular spaces (Kuswanto et al., 2012). Previous studies have also found that the diffusivity of the thalamic radial tract in adults with histories of childhood trauma is different from that in adults without histories of childhood trauma (Alyousefi-van Dijk et al., 2021; Lim et al., 2019; Meinert et al., 2019; Tendolkar et al., 2018). For example, Meinert et al. (2019) found in two cohorts that a history of childhood trauma was associated with reduced FA in the sub-frontal occipital fasciculus, leptomeningeal fasciculus, thalamic radiation, corticospinal tract, longitudinal fasciculus, cingulate fasciculus, and corpus callosum. Additionally, emotional neglect was the strongest predictor of the decline in fasciculus FA in one of the cohorts. However, their participants might have experienced multiple subtypes of childhood trauma simultaneously and were not assessed on whether they met the diagnostic criteria for each subtype (where the CEN scores in the health control group were 9.15 ± 3.77). In the present study, except for the CEN score, which was above diagnostic criteria, other subtypes of childhood trauma scored below diagnostic criteria. This ensures that the CEN group is predominantly affected by emotional neglect rather than other trauma subtypes. We, therefore, infer that the findings observed in previous studies, namely the altered integrity of thalamic radial bundle in adults with childhood trauma, are more likely to be specifically related to CEN, one of the childhood traumas.

Anatomically, the thalamic radiation links the dorsomedial nucleus of the thalamus to the prefrontal cortex via the anterior limb of the internal capsule (Coenen et al., 2012; Zhang et al., 2023). Functionally, it serves to transmit sensory and motor information to the precentral and postcentral cortices (Wycoco et al., 2013). Meanwhile, both the prefrontal cortex and the thalamus are core regions of emotional circuits, and the coordinated activity of these circuits requires maturation of the components and improved integration of the connections (Birnie & Baram, 2022). This suggests that reduced thalamic radial tract integrity may be associated with poorly developed emotional circuits. Human studies have confirmed that sensory signals from the early environment strongly influence the development and functioning of emotional circuits (Luby et al., 2020). Therefore, we speculate that, for adults with CEN, limited emotional input in their early environment may inhibit the formation of myelin sheaths in the emotional loop, leading to reduced integrity of the information transmission pathway, that is, reduced FA values.

The relationship between CEN and emotional disorders has been well established. Studies have revealed that CEN experiences lead to increased negative emotions (M. J. Jin et al., 2018; Schimmenti et al., 2015), as well as mood dysregulation (Hong et al., 2018; Huh et al., 2017). Evidence from neural science also suggests the thalamic radial tract is strongly associated with manifestations related to emotional dysfunction (Coenen et al., 2012; Lai & Wu, 2014; Spalletta et al., 2013). Coenen et al. (2012) identified a neural pathway consisting of the medial forebrain bundle and the anterior thalamic radiation that influences emotion regulation through a reward–punishment circuit and maintains a dynamic balance between positive/negative affective states. Spalletta et al. (2013) found that subclinical apathy was negatively correlated with FA in the bilateral thalamus, anterior thalamic radiations, greater magnocellular forceps, and the corona radiata in healthy female individuals. Lai and Wu (2014) found that patients with major depression had lower FA values than healthy controls in the left superior longitudinal bundle and the right anterior thalamic radiation.

Our results expand the study of the nervous system’s plasticity. Animal studies have shown that deprivation of environmental stimuli after birth can lead to severe physiological defects, such as visual cortex impairment after deprivation of vision (Fagiolini et al., 1994; Frégnac & Imbert, 1978). In contrast, animals in enriched environments had substantial increases in cortical thickness and weight, dendritic arborization, the number of dendritic spines, synaptic density, and postsynaptic thickening, which occur in multiple regions of the brain (Mohammed et al., 2002). Using DTI imaging, human studies have also found abnormal diffusion coefficients (FA) for white matter structures on the visual system in patients with early blindness, suggesting that early visual deprivation leads to the reorganization of white matter structures in the brain and affects the normal maturation of the nervous system (Kupers & Ptito, 2014; Lao et al., 2015; Noppeney et al., 2005). CEN is somewhat of an emotional deprivation. Our results suggest that not only does the deprivation of physical stimuli affect individual brain development, but the deprivation of emotional stimuli may also affect individual neurodevelopment, particularly in the right thalamic radiation.

Inevitably, there are some limitations to the present study, which call for a cautious interpretation of these results. First, while the DTI-based automated fiber-tracking method in this study is validated and reliable (Daood et al., 2025; Yeatman et al., 2012, 2014), it struggles with complex fiber configurations like crossovers or branching, potentially reducing sensitivity to microstructural changes across multiple bundles. This limitation might explain why we observed intergroup differences in only one bundle. Future research should investigate advanced methods like fixel-based analysis, which can effectively disentangle crossing and branching fiber populations within each voxel (Dhollander et al., 2021), to better understand white matter structure in children trauma. Second, although DTI, AFQ, and SVMs have all been successfully applied to small-sample studies (Payabvash et al., 2019; Wee et al., 2011), and our three-stage screening process and stringent inclusion criteria further ensured the representativeness of the sample and the reliability of the results, the relatively small number of participants inevitably limited the generalizability of our findings. Finally, we chose a sample of young adults whose white matter development was approaching peak maturity. This choice facilitated the exploration of the long-term outcomes of CEN’s effects on brain white matter. However, it also made it difficult to account for temporal variations. Future studies should validate this study’s results by longitudinally tracking the effect of CEN in different age groups. Moreover, future studies could use emotional tasks and brain functional imaging techniques to more deeply reveal the relationship between CEN and emotional circuits.

## 5. Conclusions

This study combined questionnaires and the diffusion imaging technique to examine the effects of CEN only on the white matter structure of young adults. It was found that the CEN group had reduced FA values in the right thalamic radiation compared to the control group, indicating a decrease in white matter integrity. Reduced white matter integrity in the thalamic radiation bundle may reflect impaired emotional circuits, which in turn increases the risk of psychiatric disorders. Our results enrich the theoretical foundation of “experience shapes the brain”.

## Data Availability

De-identified and pre-processed data will be made available on reasonable request.
